# Genome-level homology and phylogeny of *Shewanella *(Gammaproteobacteria: lteromonadales: Shewanellaceae)

**DOI:** 10.1186/1471-2164-12-237

**Published:** 2011-05-12

**Authors:** Rebecca B Dikow

**Affiliations:** 1 Committee on Evolutionary Biology, The University of Chicago, Chicago, IL, USA; 2 Division of Fishes, The Field Museum of Natural History, Chicago, IL, USA

## Abstract

**Background:**

The explosion in availability of whole genome data provides the opportunity to build phylogenetic hypotheses based on these data as well as the ability to learn more about the genomes themselves. The biological history of genes and genomes can be investigated based on the taxomonic history provided by the phylogeny. A phylogenetic hypothesis based on complete genome data is presented for the genus *Shewanella *(Gammaproteobacteria: Alteromonadales: Shewanellaceae). Nineteen taxa from *Shewanella *(16 species and 3 additional strains of one species) as well as three outgroup species representing the genera *Aeromonas *(Gammaproteobacteria: Aeromonadales: Aeromonadaceae), *Alteromonas *(Gammaproteobacteria: Alteromonadales: Alteromonadaceae) and *Colwellia *(Gammaproteobacteria: Alteromonadales: Colwelliaceae) are included for a total of 22 taxa.

**Results:**

Putatively homologous regions were found across unannotated genomes and tested with a phylogenetic analysis. Two genome-wide data-sets are considered, one including only those genomic regions for which all taxa are represented, which included 3,361,015 aligned nucleotide base-pairs (bp) and a second that additionally includes those regions present in only subsets of taxa, which totaled 12,456,624 aligned bp. Alignment columns in these large data-sets were then randomly sampled to create smaller data-sets. After the phylogenetic hypothesis was generated, genome annotations were projected onto the DNA sequence alignment to compare the historical hypothesis generated by the phylogeny with the functional hypothesis posited by annotation.

**Conclusions:**

Individual phylogenetic analyses of the 243 locally co-linear genome regions all failed to recover the genome topology, but the smaller data-sets that were random samplings of the large concatenated alignments all produced the genome topology. It is shown that there is not a single orthologous copy of 16S rRNA across the taxon sampling included in this study and that the relationships among the multiple copies are consistent with 16S rRNA undergoing concerted evolution. Unannotated whole genome data can provide excellent raw material for generating hypotheses of historical homology, which can be tested with phylogenetic analysis and compared with hypotheses of gene function.

## Background

*Shewanella *is a genus of marine and freshwater gram-negative Gammaproteobacteria within the monogeneric family Shewanellaceae Ivanova et al., 2004. While members of *Shewanella *have been recognized since 1931 (e.g. *Achromobacter putrefaciens *Derby and Hammer 1931 now *Shewanella putrefaciens*), the genus *Shewanella *has only been recognized with its present name since 1985 [1] and 39 of the 52 currently recognized species have been described since 2000 [2]. There are also multiple strains that are commonly studied but have not been given a proper name (some of these have been included below and will be referred to by their strain number). Members of *Shewanella *have been described from diverse habitats, including deep cold-water marine environments to shallow Antarctic Ocean habitats to hydrothermal vents and freshwater lakes (see Table 1[1,3-21]). *Shewanella *has been of great interest due to the ability of its species to convert heavy metals and toxic substances (e.g. iron, sulfur, uranium) into less toxic products by using them as electron acceptors in certain respiratory situations, making them of interest for environmental clean-up (e.g. iron, sulfur: [22]; uranium: [23]). To this end, 19 genomes have been fully sequenced and deposited on GenBank as of 2009. Annotations suggest that species possess approximately 5,000 genes and have genomes of approximately 5 Mbp (details in Table 1).

**Table 1:** Taxon table and Mauve results

			LCBs present in all taxa			LCBs present in subsets of taxa		
Taxon	RefSeq accession	Genome length	bp covered	% of genome	# of genes	bp covered	% of genome	locality
*Aeromonas hydrophila *ATCC 7966	NC_008570	4,744,448	1,132,632	23.9	1,096	1,971,555	41.6	Fresh and marine [3]
*Alteromonas macleodii *deep ecotype	NC_011138	4,412,282	1,209,099	27.4	1,223	1,607,063	36.4	Mediterranean Sea [4]
*Colwellia psychrerythraea *34H	NC_003910	5,373,180	1,272,052	23.7	1,279	2,130,197	39.6	Arctic sediments [5]
*Shewanella amazonensis *SB2B	NC_008700	4,306,142	1,312,077	30.5	1,279	3,228,613	79.4	Baltic Sea 120 m [6]
*Shewanella baltica *OS223	NC_011663	5,145,902	1,596,790	31.0	1,504	4,924,714	95.7	Baltic Sea 240 m [7-9]
*Shewanella baltica *OS155	NC_009052	5,127,376	1,515,912	29.6	1,469	4,778,750	93.2	Baltic Sea 240 m [7-9]
*Shewanella baltica *OS185	NC_009665	5,229,686	1,579,905	30.2	1,511	4,997,928	95.6	Baltic Sea 240 m [7-9]
*Shewanella baltica *OS195	NC_009997	5,347,283	1,572,889	29.4	1,496	5,038,451	94.2	Baltic Sea 240 m [7-9]
*Shewanella denitrificans *OS217	NC_007954	4,545,906	1,318,644	29.0	1,244	2,749,058	60.5	Baltic Sea deep [10]
*Shewanella frigidimarina *NCIMB 400	NC_008345	4,845,257	1,306,074	27.0	1,253	3,068,494	63.3	North Sea [11]
*Shewanella halifaxensis *HAW-EB4	NC_010334	5,226,917	1,469,687	28.1	1,407	4,727,461	90.4	Nova Scotia [12]
*Shewanella loihica *PV-4	NC_009092	4,602,594	1,339,527	29.1	1,306	3,377,749	73.4	Hawaii, USA [13]
*Shewanella oneidensis *MR-1	NC_004347	4,969,803	1,525,080	30.7	1,479	4,360,652	87.7	Oneida Lake, USA [14]
*Shewanella pealeana *ATCC 700345	NC_009901	5,174,581	1,485,394	28.7	1,424	4,573,456	88.4	Atlantic Ocean [15]
*Shewanella piezotolerans *WP3	NC_011566	5,396,476	1,485,147	27.5	1,482	4,252,921	78.8	Pacific Ocean [16]
*Shewanella putrefaciens *CN-32	NC_009438	4,659,220	1,596,811	34.3	1,559	4,351,757	93.4	Fresh and marine [1,17]
*Shewanella sediminis *HAW-EB3	NC_009831	5,517,674	1,477,164	26.8	1,391	4,444,654	80.6	Nova Scotia [18]
*Shewanella sp*. ANA-3	NC_008577	4,972,204	1,494,516	30.1	1,394	4,647,695	93.5	Woods Hole, USA [19]
*Shewanella sp*. MR-7	NC_008322	4,792,610	1,478,293	30.8	1,392	4,564,063	95.2	Black Sea [14]
*Shewanella sp*. MR-4	NC_008321	4,706,287	1,478,804	31.4	1,499	4,567,658	97.1	Black Sea [14]
*Shewanella sp*. W3-18-1	NC_008750	4,708,380	1,578,878	33.5	1,517	4,443,111	94.4	Coastal Pacific [20]
*Shewanella woodyi *ATCC 51908	NC_010506	5,935,403	1,503,207	25.3	1,476	4,442,801	74.9	Mediterranean Sea [21]
								
# of LCBs			243			3,004		
alignment length			3,361,015			12,456,624		

The goal of the study presented here is to investigate how we can use whole genome data, not only to build a tree but to inform us of gene and genome history by comparing the hypothesis of historical homology supported by the phylogenetic hypothesis to what is known about gene function. There is a computational interest in the ability to build large trees, both in number of taxa and number of characters, e.g. [24,25]. The biological history of genes and genomes can be investigated based on the taxomonic history of the bearers of these characters. This goes further than just the prediction of function of uncharacterized genes, but also includes the potential to track changing function over gene history and finding up- or down-stream segments of co-evolving DNA. Eisen and Fraser highlighted many of these goals when they introduced the term "phylogenomics" [26]. While these goals are broad and ambitious, it is the hope that the present study represents a step in this direction.

The presented approach also represents a shift for phylogenetic systematics, in which historically one has generally known all the characters of interest very well and perhaps had a well-formed opinion about their history based on a lifetime of knowledge about their distribution and subtle variations. Even with molecular characters in the form of one or a few genes, even with many taxa, one gets to know the 'reliable' parts of an alignment and often memorizes the DNA sequence after having sequenced and edited the same marker for several years. The approach presented here proposes a new perspective which is obligated by the new kinds of data being gathered, particularly those from next-generation and shotgun sequencing, which generate millions of nucleotide base-pairs (bp) as opposed to thousands. Primary homology (*sensu *dePinna, [27]) must be determined in an automated fashion given the vast amount of data and the few character states of nucleotide data. The phylogenetic tree becomes an intermediate point - it is built based on hypotheses of primary homology, which it tests, and then is used as a framework for optimizing the character states and looking back to functional gene annotations to begin to answer questions about gene and genome history. Polymerase chain reaction (PCR) primers can provide hypotheses of primary homology, as amplifications using primers target conserved flanking regions, which provide a sufficient level of confidence that the 'same' regions are being sequenced. With next-generation sequencing, we have no such sense of location (particularly with bacteria), as we expect rearrangement of genes or other genomic segments over evolutionary history [28-31]. Annotations can provide information about the function of genes and the location of open reading frames, but these may not lead us to historical homology and will miss much of the homology present in the genome, for example among genes of altered function or non-coding DNA.

A second goal of this study is to present an example of comparative genomics for a closely related taxon that is densely sampled. This helps to avoid some of the downsides of comparative genomics when few, disparate taxa are compared: leaps of faith with character homology and trivial phylogenetic topologies. Finally, as an attempt to begin the investigation of gene history, 16S rRNA is presented as a test case. 16S rRNA was chosen because it is the marker that has defined prokaryote taxonomy for the past 30 years [32]. While it is not used without some skepticism, e.g. [33,34], the way that primary homology is generated here provides an opportunity to test our assumptions about 16S rRNA. This manuscript will address the outlined points in the following sections: Mauve primary homology [35], genome trees, subset trees, and 16S rRNA investigation.

## Methods

For all taxa of *Shewanella *for which the genome sequence has been completed and deposited on GenBank, 16 species and 3 additional strains of one species and for three outgroup taxa, unannotated genome sequences were downloaded (strain and accession numbers in Table 1). Seven gene loci were also downloaded individually for all 22 taxa from the same genome accession numbers. The genes are *gyrB *(DNA gyrase subunit B), *rpoA *(DNA directed RNA polymerase subunit alpha), *recA *(recombinase A), *topA *(DNA topoisomerase I), *mreB *(rod shape-determining protein), *gapA *(glyceraldehyde-3-phosphate dehydrogenase), and *atpA *(ATP synthase F1 subunit alpha). Those species for which only one strain is included will hereafter be referred to by only their species name. For *Shewanella baltica*, for which multiple strains have been sampled, I will refer to the strain numbers throughout. Annotations were also downloaded from RefSeq (GenBank's Reference Sequence Collection, 2009). The sampled genomes range in size from 4,306,142 nucleotide base-pairs (bp) and 3,785 genes (*Shewanella amazonensis*) to 5,935,403 bp and 5,096 genes (*Shewanella woodyi*). All of the below analyses were performed on a 2.8 GHz Quad-Core MacPro with 20 GB RAM.

### Mauve primary homology

Sequences were submitted to a genome alignment using Progressive Mauve [35]. This program has been used previously on fairly closely related members of Gammaproteobacteria [36]. Traditional multiple sequence alignment cannot be used on complete genome sequences because significant rearrangement of genes or fragments has been shown to occur over evolutionary history [28-31]. Mauve addresses this issue by finding locally collinear blocks (LCBs), or contiguous segments of sequence within which there has not been rearrangement, but within a longer sequence that may have been subject to rearrangement events. The default parameters in Mauve were used. Mauve finds anchor points of similarity and then extends these matches outward. A single LCB becomes two when a sequence segment is found somewhere else in the genome for one of the taxa, meaning that a rearrangement has occurred. Mauve does not allow one sequence fragment to be homologous to more than one fragment in another species. CGView [37] has been used to plot the genomic location of LCBs found common to all species and to project gene annotations onto LCB alignments. Resulting LCBs were submitted to standard multiple sequence alignment with MAFFT [38]. Mauve output XMFA files include the base-pair ranges (e.g. bp 1-10,000) of each LCB for each taxon. The base-pair locations were used along with annotations to assess which genes were present in each LCB. At the suggestion of a reviewer, Mauve was also run with a single outgroup taxon, *C. psychrerythraea*, to compare the influence of one vs. more than one outgroup on the percent of the genome covered by the Mauve alignment.

### Genome trees

Aligned LCBs were concatenated and submitted for phylogenetic analysis with parsimony in TNT [39], maximum likelihood in RaxML v. 7.0.4 [40], and Neighbor Joining [41] in Geneious Pro 4.8.3 [42]. Long alignments are challenging for phylogenetic tree building programs as many of these programs have a limit on the number of input characters or cannot access enough RAM to read the alignment. Those programs mentioned above were able to read the data analyzed here. The TNT commands consisted of 1000 builds with SPR and TBR followed by 1500 replicates of ratchet and tree fusing [43,44]. RaxML was run under the GTRGAMMA model of nucleotide substitution. Only a single bootstrap replicate was attempted at a time, as this seemed to be the computational limit, at least for the used workstation, as any additional searching caused an error. Neighbor Joining was run with Jukes-Cantor, Tamura-Nei, and HKY genetic distance models in three separate analyses. Bootstrap resampling was attempted in TNT, with 5,000 pseudoreplicates. As an additional measure of support, trees were built from individual LCBs and were used to score whether nodes in the genome tree were also present in the individual LCB trees. Gaps were initially treated as a fifth state in TNT but all genome-wide data-sets were also reanalyzed with gaps treated as missing data. When not specified, the TNT analyses refer to those in which gaps were treated as a fifth state.

### Subset trees

In order to compare the phylogenetic signal from the entire genome to signals from subsets of the genome, three courses of action were taken. The first asks the question whether localized signal is congruent with whole genome signal. To this end, phylogenetic hypotheses were built for individual LCBs, as above, in TNT. Second, in order to address whether the number of characters has a direct result on the topology, scripts using BioPerl were used to randomly sub-sample the nucleotide alignment and produce data-sets of varying size, 20,000 bp, 100,000 bp, 500,000 bp and 1,000,000 bp, which were submitted to the same phylogenetic treatment as above. Finally, a seven-gene analysis was attempted in TNT, as above, to compare a traditional phylogenetic analysis to the genome-level analysis that was the main goal of the study.

### 16S rRNA investigation

16S rRNA copies were tracked throughout the Mauve LCBs in order to establish for which of the 16S rRNA copies Mauve made an hypothesis of homology. All copies of 16S rRNA for all taxa (taxa have 8-11 copies each, according to annotations, for a total of 200 terminal branches for the 22 taxa), whether or not present in an LCB, were extracted from their respective annotated genome files, aligned with MAFFT, and submitted for phylogenetic analysis in TNT and RaxML, as above.

## Results

### Mauve primary homology

**Figure 1:**
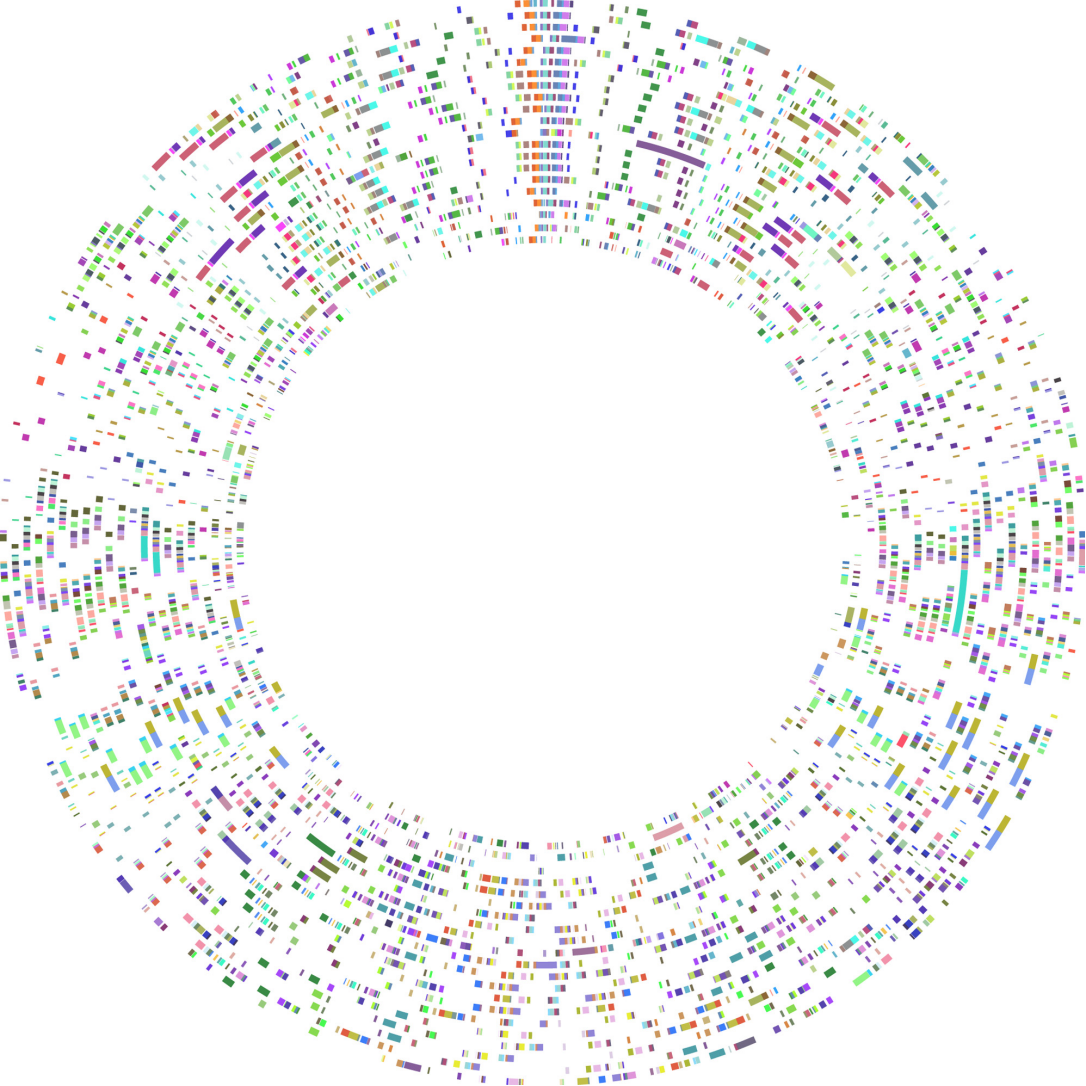
**Circular 243 LCB plot**. LCB location plotted in CGView. Each circle represents a genome. From the innermost circle: *A. hydrophila, Al. macleodii, C. psychrerythraea, S. amazonensis, S. loihica, S. baltica OS 155, S. baltica 223, S. baltica 185, S. baltica OS 195, S. putrefaciens, S. sp. W3-18-1, S. oneidensis, S. sp. ANA-3, S. sp. MR-7, S. sp. MR-4, S. woodyi, S. sediminis, S. piezotolerans, S. halifaxensis, S. pealeana, S. denitrificans, S. frigidimarina*. Colored blocks represent LCBs, blocks of the same color represent the same numbered LCB.

Mauve results are summarized in Table 1. Figure 1 is a CGView plot, which shows the genomic locations of those LCBs present for all taxa. Each circle represents a taxon (genome) and the colored blocks represent the individual LCBs. Blocks of the same color are putatively homologous LCBs. White space represents the parts of the genome within which Mauve did not find homology among all taxa sampled. Mauve ran for approximately 7 days and 243 LCBs were found common to all taxa. The length of individual LCBs after DNA sequence alignment ranged from 645 bp to 129,020 bp and the number of genes per LCB ranged from zero to 30 and the mean was 6.07 genes per LCB for *S. woodyi*, for example. When added together, these 243 all-taxa LCBs represented a range from 23.7 to 34.3% of the genomes and 1096 and 1559 genes (as per annotations from RefSeq) along with non-coding DNA (Table 1). For *S. sp. MR-7*, for example, there are 218,403 non-coding nucleotide base-pairs included, 14.8% of the unaligned nucleotide base-pairs. The concatenated alignment was 3,361,015 bp (hereafter referred to as the 3 Mbp alignment). The sequence identity of the alignment is 48.7%. If we also consider those LCBs shared among subsets of taxa (at least 2) that contained >100 bp, there were 3004 LCBs, which represented 36.4 to 97.1% of the genomes (Table 1). The concatenated alignment of these 3004 LCBs was 12,456,624 bp (hereafter referred to as the 12 Mbp alignment). When the only outgroup taxon considered is *C. psychrerythraea*, there were 325 LCBs and the concatenated alignment was 4,604,291 bp. *C. psychrerythraea *had 1,690,088 unaligned bp present in the Mauve alignment when it was the only outgroup, as compared to 1,272,052, when the other two outgroups were also included.

### Genome trees

**Figure 2:**
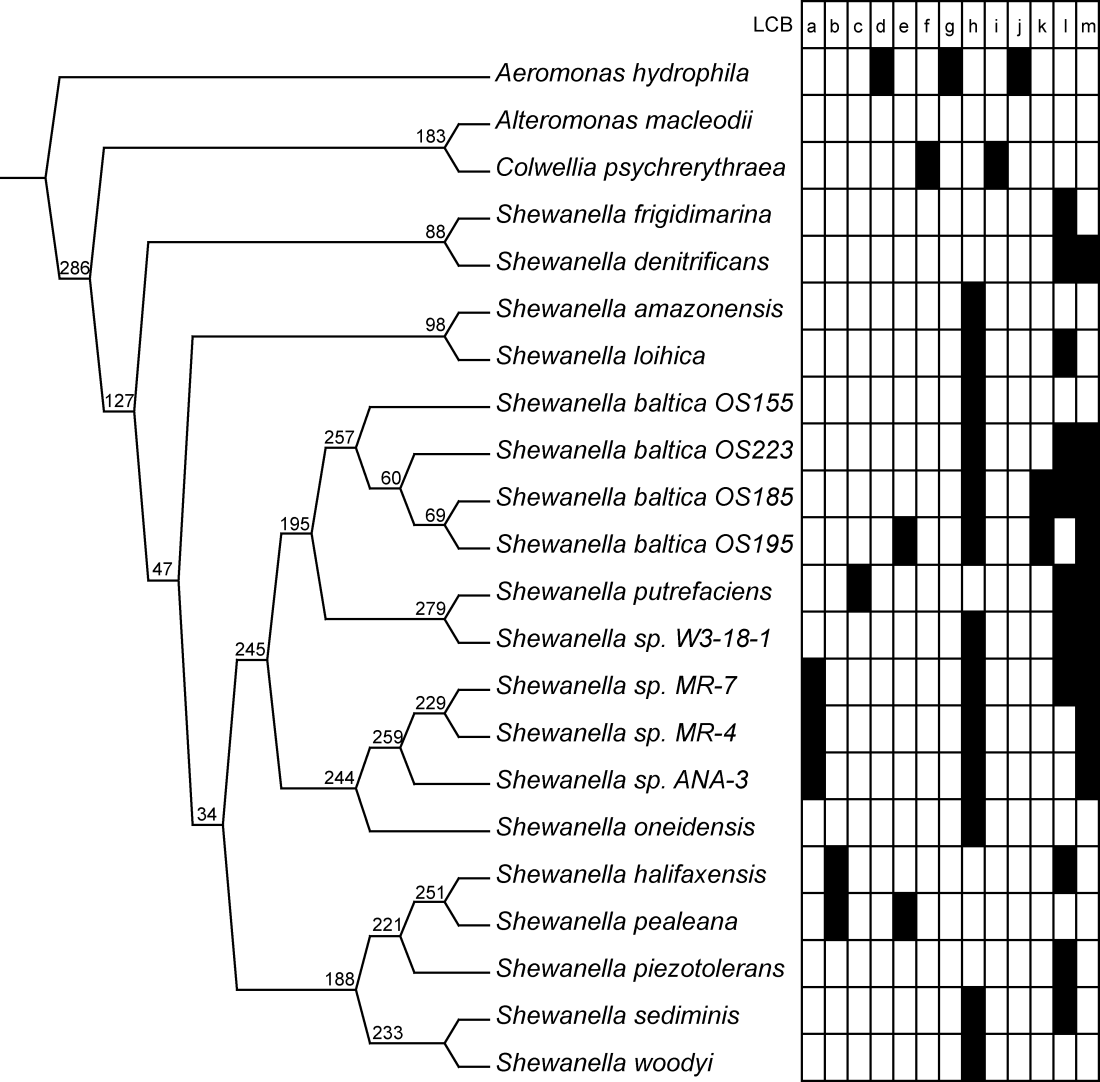
**Genome tree**. Most parsimonious/Most likely tree from TNT and RaxML, respectively. Number of TNT trees from individual LCBs possessing a particular node shown above the branches. LCBs with 16S rRNA copies shown in grid. Filled boxes have a copy of 16S rRNA for that particular taxon. LCBs have been labeled with letters representing their numbered location in the genome: a = 4, b = 7, c = 19, d = 27, e = 37, f = 58, g = 60, h = 79, i = 142, j = 197, k = 219, l = 222, m = 231.

**Figure 3:**
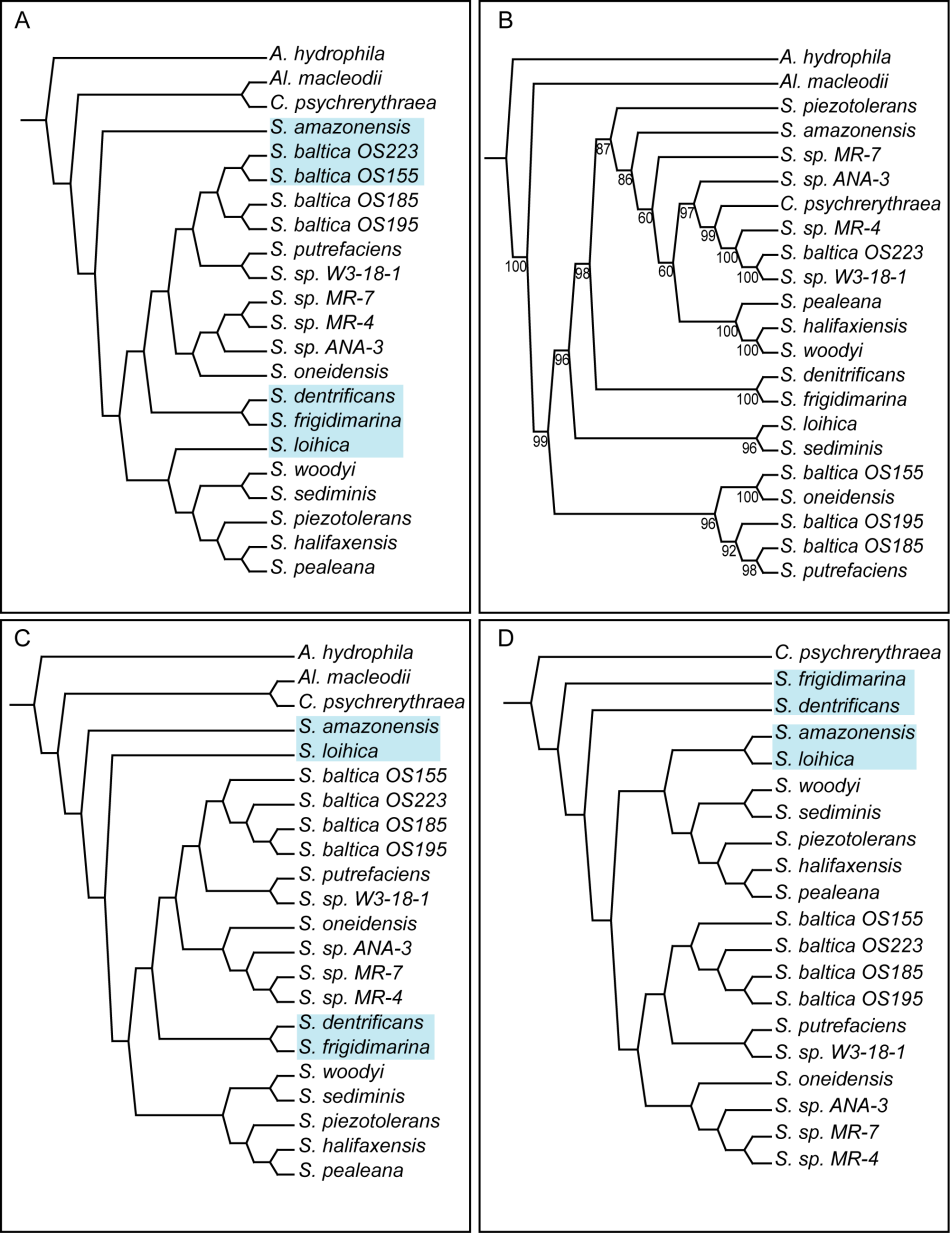
**Neighbor Joining, seven-gene, gaps-as-missing, and single-outgroup trees**. A: Neighbor Joining topology for both 3 Mbp and 12 Mbp alignments; B: Most parsimonious tree from TNT based on seven gene loci, with boopstrap resampling frequencies below the branches; C: Most parsimonious tree from TNT when gaps in 3 Mbp alignment are treated as missing; D: Most parsimonious tree from TNT when *C. psychrerythraea *is the only outgroup taxon. Taxa with variable placement are highlighted to show topology differences in A, C, and D.

For the 3 Mbp alignment, that was based on the 243 all-taxa LCBs, the TNT (gaps as fifth state) and RaxML trees have the same topology (shown in Figure 2 - hereafter referred to as the 'genome tree'), while the NJ tree does not (Figure 3A). The only reason NJ was attempted was to investigate the idea that when there are so many data, phylogenetic hypotheses might converge on those constructed from similarity only. In this case, the topologies are not the same. All three NJ genetic distance models produced the same topology. The TNT tree for the 3 Mbp alignment had 7,875,803 steps. Multiple individual RaxML runs all produced the same topology. The likelihood score was -860351.367382. The 12 Mbp alignment and 3 Mbp alignment gave the same topology in TNT, which is not unexpected given that the 3 Mbp alignment is just a subset of the 12 Mbp alignment. The 12 Mbp alignment produced a tree of 29,512,322 steps in TNT but a tree was not found in RaxML; the program terminated with an error. The NJ trees for the 12 Mbp alignment had the same topology as the NJ trees for the 3 Mbp alignment (Figure 3A). When gaps are treated as missing data for the 3 Mbp alignment in TNT (Figure 3C), *S. amazonensis *and *S. loihica *are no longer sister, and the placement of *S. denitrificans *+ *S. frigidimarina *is much more derived. This tree had 4,274,327 steps. *Shewanella *formed a monophyletic group in every case, however. Bootstrap values from TNT are not shown for the genome tree, as they are 100% for every node. Above the branches in Figure 2 is the number of trees built from the 243 individual LCBs (from the 3 Mbp alignment) that contain that particular node present in the genome tree. The analysis in which *C. psychrerythraea *is the only outgroup taxon included is shown in Figure 3D. The most parsimonious tree had a length of 10,573,382 when gaps are treated as a fifth state. The same topology resulted when gaps were treated as missing, but the tree cost was 5,753,742 steps. Trees have been deposited in TreeBASE and may be accessed at http://purl.org/phylo/treebase/phylows/study/TB2:S11219.

### Subset trees

On the trees built from the 243 individual LCBs in TNT, for which there were 286 most parsimonious trees (MPTs), none had the topology of the genome tree with gaps as fifth state. There are 285 unique topologies represented in these 286 trees. None of these 286 trees has the topology of the gaps as missing TNT analysis either. While the individual LCB trees cannot be referred to as gene trees *per se*, as the vast majority of LCBs contain more than one gene as well as non-coding DNA, the phenomenon of parts of a data-set not producing the same phylogenetic signal, is comparable. To address whether it was an issue of number of base-pairs versus different localized signals concentrated in different contiguous parts of the genome (*i.e*. each LCB), I used the data-sets generated with BioPerl scripts, that randomly selected nucleotide columns from the 3 Mbp alignment and separately for the 12 Mbp alignment. These data-sets consisted of 20,000 bp, 100,000 bp, 500,000 bp and 1,000,000 bp for each of the original alignments. When I performed phylogenetic analyses on these data-sets, all produced the same topology as the phylogenetic (TNT and RaxML) genome tree; the same topology that was not found for any of the 243 single-LCB data-sets. For the seven-gene dataset, genes were aligned separately in MAFFT and then concatenated, resulting in an alignment of 13,009 bp. The topology resulting from this analysis is shown in Figure 3B with bootstrap resampling frequencies shown below the branches.

### 16S rRNA investigation

The next step was to associate the annotation, or list of open reading frames (ORFs) and their locations, with the list of LCB boundaries for each species. Of initial interest was tracking the multiple copies of 16S rRNA within these taxa and assessing their presence within the LCBs. There were 13 LCBs that contained a copy of 16S rRNA for at least one species. These are shown in the grid next to the tree in Figure 2. The LCBs are 4 (*S. sp. MR-7*, *S. sp. MR-4*, *S. sp. ANA-3*), 7 (*S. halifaxensis*, *S. pealeana*), 19 (*S. putrefaciens*), 27 (*A. hydrophila*), 37 (*S. baltica OS195*), 58 (*C. psychrerythraea*), 60 (*A. hydrophila*), 79 (*S. amazonensis*, *S. loihica*, *S. baltica OS155*, *S. baltica OS223*, *S. baltica OS 185*, *S. baltica OS195*, *S. sp. W3-18-1*, *S. sp. MR-7*, *S. sp. MR-4*, *S. sp. ANA-3*, *S. oneidensis*, *S. sediminis*, *S. woodyi*), 142 (*C. psychrerythraea*), 197 (*A. hydrophila*), 219 (*S. baltica OS185*, *S. baltica OS195*), 222 (*S. frigidimarina*, *S. denitrificans*, *S. loihica*, *S. baltica OS223*, *S. baltica OS185*, *S. putrefaciens*, *S. sp. W3-18-1*, *S. sp. MR-7*, *S. halifaxensis*, *S. piezotolerans*, *S. sediminis*), 231 (*S. denitrificans*, *S. baltica OS223*, *S. baltica OS185*, *S. baltica OS195*, *S. putrefaciens*, *S. sp. W3-18-1*, *S. sp. MR-7*, *S. sp. MR-4*, *S. sp. ANA-3*). The first obvious pattern is that, while all copies of 16S rRNA are homologous (derived from a single 16S rRNA ancestral copy), there was no single copy of 16S rRNA aligned by Mauve across all taxa, which would indicate the possibility of orthology. When the LCBs present in subsets of taxa were considered, and the subset of interest is all *Shewanella *species, there was also no single 16S rRNA copy aligned across all *Shewanella *taxa. It must also be noted that it is possible that there is a single orthologous copy that Mauve is unable to recover.

**Figure 4:**
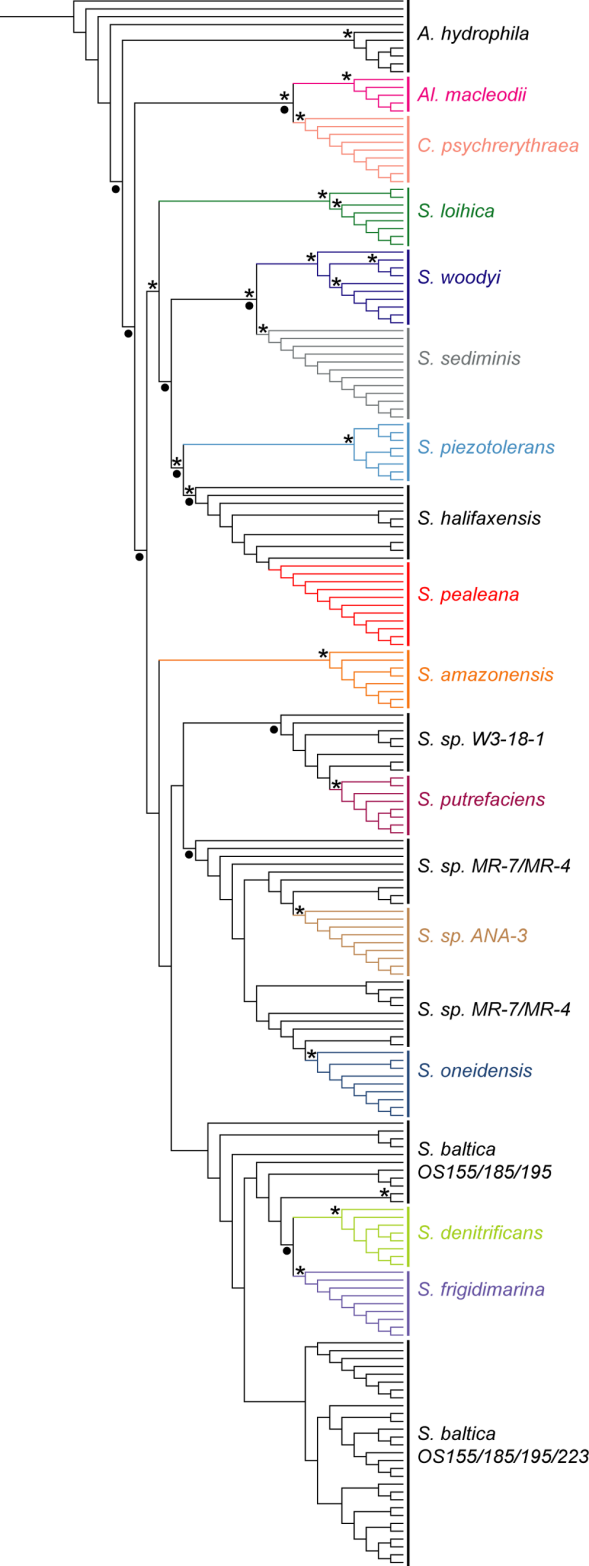
**Tree of all copies of 16S rRNA**. Monophyletic clades and colored; paraphyletic and polyphyletic clades are black. Circles below the branches indicate topological congruence with the genome tree. Stars above branches indicate bootstrap resampling frequencies above 75%.

When all 16S rRNA copies were extracted from annotated genomes and subjected to multiple sequence alignment and phylogenetic analysis, for a number of species, two 16S rRNA copies within a particular taxon were identical, but there were never any copies identical among different species. For *S. baltica *strains, however, there were instances where one strain had a copy identical to a copy in another strain. The results of the phylogenetic analysis of all copies of 16S rRNA were, from TNT, a MPT with 1275 steps (Figure 4). Out of the 1584 characters, 338 were parsimony informative. Topological differences between the TNT tree and that produced by RaxML will be addressed in the discussion.

## Discussion

### Mauve primary homology

**Figure 5:**
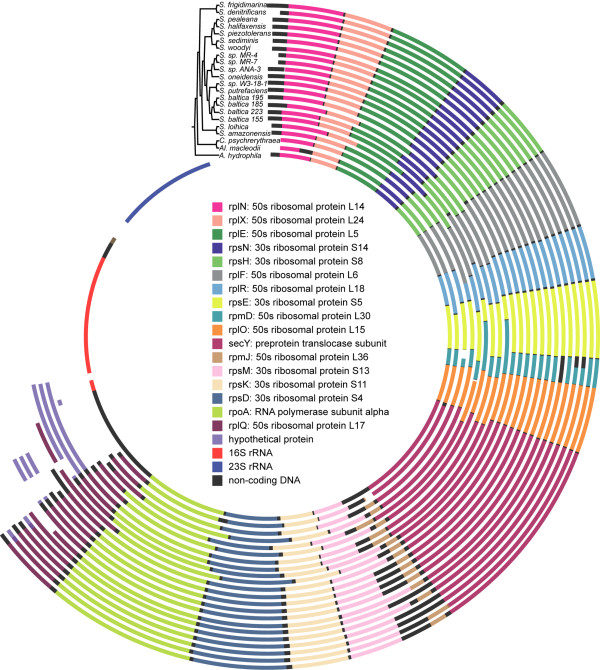
**LCB 27**. Gene annotation projected onto LCB sequence alignment and plotted in CGView for LCB 27. This alignment is 13,022 bp.

The approach Mauve takes to assigning primary homology does not take function, gene boundaries, or ORFs into account *a priori*. Mauve allows each sequence fragment to be homologous to only one other fragment. Also, when we consider only the LCBs present in all taxa, we avoid fragments that might be recently laterally transferred, because these would not be present in all taxa. Since Mauve does not consider annotation *a priori*, one can compare the Mauve alignment to annotation without following circular reasoning to ascertain which genes are aligned to one another (putative gene homologies). Questions or hypotheses about probable function for unknown genes and history of regions of conflicting function can now be studied. Figure 5 illustrates this approach. For LCB 27, which consists of 13,022 aligned nucleotide base-pairs, the gene annotations have been projected onto the alignment. What is obvious from this plot is that in general, the gene boundaries match up well and the gene content of LCB 27 is fairly consistent across the taxa sampled. But there are areas of interest, for example *rpmD *(or 50S ribosomal protein L30) is present in most taxa, but absent in *S. sediminis*, filled instead by non-coding DNA. This pattern is consistent with multiple possible explanations, including (1) poor annotation, (2) sequencing error, (3) this gene has become inactivated or degraded significantly enough that it is not recognized by annotation, or (4) this gene has really been deleted from the genome. Furthermore, the length variation in particular genes is put into context and we can see perhaps from what kind of material (non-coding or gene) they have been transformed. The length variation might not only indicate *de novo *insertions of material, but conversion from a neighboring gene. This possibility is illustrated in *rpsE *and *rpmD*, where *rpmD *spills into *rpsE *for two *S. baltica *strains (Figure 5).

The allowance for variation within an LCB for base-pair length and number of genes among different taxa is how the estimates of primary homology considered by Mauve differ from previous studies: they do not require that a gene, which might look functionally the same, be homologous (aligned) across all taxa simply because it is present in these taxa. This is key in our beginning to understand gene histories and how these histories interact with the functional roles of genes. The homologies among fragments of non-coding DNA are also important, because if these kinds of analyses are extended to eukaryotes, which have vastly more non-coding DNA, we will be looking for ways to include these data and not be limited to evidence of history only from genes, which might have conflicting histories.

As can be seen in Table 1 in the all-taxa LCB data-set, the coverage by LCBs among included taxa ranges between 23-34%. In the subset taxa LCB data-set, one can track the coverage by phylogenetic position, with the innermost nodes having the most complete coverage. This coverage (Subset taxa LCBs) ranges from 36-97%. Outgroup taxa have a lower percentage of their genome covered by LCBs than do ingroup taxa. This pattern would be expected if the outgroups are indeed more distant relatives than any of the ingroup taxa. The inclusion of outgroups in a Mauve analysis decreases the LCB coverage, because they are more distantly related, but is essential to polarizing the characters obtaining the optimal phylogenetic topology. The reason three outgroup taxa were included was because there had not been a previous study showing that *Alteromonas *and *Colwellia *were indeed basal to *Shewanella*. One could not assume that *Shewanella *was monophyletic *a priori*; the seven-gene analysis (Figure 3B) finds a non-monophyletic *Shewanella*. To address a reviewer comment that using a single outgroup would increase the amount of data considered in the phylogenetic analysis, however, another Mauve run was attempted with a single outgroup (*C. psychrerythraea*). This species was chosen because it was found to be the most closely related to *Shewanella*, and therefore would cause the greatest increase in data if the other outgroups were not included. If just the most basal outgroup was included, one would not expect much of a change in genome coverage. The initial analysis, in which all three outgroup taxa are included, allows one to make the most *a posteriori *claims about homology for the broadest range of taxa. The topological differences are discussed below.

### Genome trees

The number of parsimony informative characters in the 3 Mbp alignment (26.48%) was within the range of many molecular studies based on one or a few genes, e.g. [45,46]. This fact provides a sense of confidence that Mauve was finding real, or at least likely, homologies. This percentage ranged from 1.4% to 53.6% when LCBs were compared to each other. If the percentage of parsimony informative characters had been significantly higher, I might be concerned that non-homologous and non-similar sequences were being aligned. In the RaxML analysis, gaps were treated as missing. Gaps were treated as a fifth state as the default for the parsimony (TNT) analyses because gaps do represent evolutionary events. The nature of the Mauve analysis, in my opinion, lends itself to the treatment of gaps as fifth-state characters because Mauve either breaks up a single LCB into two or more if homologous fragments are found in different positions in one or more genomes. If Mauve has postulated one LCB instead of two, it is then assumed that gaps represent the lack of homologous DNA sequence, not missing data. As for statistical support, values of 100% on all nodes are not particularly telling. By scoring the individual LCB trees for nodes present in the genome tree, it is clearer which nodes are the most and least robust. The relationships among strains of *S. baltica *have low support (60/286 trees), as might be expected if these have either recently diverged, or are exchanging genes. Confidence in the topology is also supported by the results of the random data-set analyses, which amount to a jackknife technique (without replacement).

When gaps are treated as missing data, as well as when *C. psychrerythraea *is the only outgroup taxon considered, there are topological differences in the placement of *S. amazonensis*, *S. loihica*, *S. denitrificans*, and *S. frigidimarina*, although *S. denitrificans *and *S. frigidimarina *are always sister. The remaining taxa retain their relationships. The node that separates *S. denitrificans, S. frigidimarina*, *S. amazonensis*, and *S. loihica *from the remaining species of *Shewanella *is the least well supported node in the tree, with 34 out of 286 individual LCB trees containing that node, indicating that it is not surprising that these four taxa are the ones that are placed differently in the various permutations of genome-wide data-sets. It is interesting that the RaxML tree and the gaps as fifth-state TNT tree have the same topology. There are multiple possible explanations for this finding. A reviewer mentioned that if genes are lost or gained in a single step, that considering gaps individually dramatically over-emphasizes the number of evolutionary events. It is also important to note, however, that by treating gaps as missing, one is ignoring those evolutionary events completely. It could be that a significant proportion of the evidence is lost in the gaps as missing analyses, but that maximum likelihood is better at compensating for multiple hits. To address the possibility that gaps, representing insertion/deletion (indel) events are overwhelming the phylogenetic signal, the number of parsimony tree steps resulting from indel events at internal nodes has been calculated to be 814,304. This number is 23.6% of all indels (the rest being at the terminal branches, autapomorphies that provide no information on branching pattern in parsimony) and 10.3% of total tree length. These calculations show that information from gaps does not make up a dominant proportion of the phylogenetic signal. It is entirely possible that the lack of indel data in the parsimony-missing data analyses leads to a different answer, but the presence of indel data in the gaps as fifth-state analysis and the ability of maximum likelihood to account for saturation give the same tree. This just argues for evidence that the genome tree (RaxML, parsimony gaps as fifth state) is the optimal tree.

For 22 taxa, there are 1.31 × 10^25 possible rooted bifurcating trees [47]. It is surprising, then, that there was an immediate convergence on the optimal topology for the 12 Mbp, 3 Mbp, and random data-sets when run in TNT. Wagner tree build plus TBR branch swapping found the optimal tree very rapidly, within a minute. No amount of ratcheting or tree fusing altered the topology or tree length. The decisiveness of these data [48] marks the difference between analyses consisting of one or a few genes and the present analysis. Just as the idea of support needs to be adjusted to accommodate whole genome analyses, so do our expectations of tree search.

A recent paper considering the systems biology of *Shewanella *based on the whole genome sequences of 10 taxa (a subset of those included here) produced a phylogeny for those 10 taxa based on 1507 single-copy orthologs identified through a combination of, "(i) protein-protein pairwise reciprocal BLAST (blastp); (ii) reciprocal protein genomic sequence best match (tblastn); and (iii) Darwin pairwise best hit" ([49], p. 15914). Their tree had the same topological relationships for those 10 taxa as the genome tree presented here. It is also interesting that the number of genes found to be single-copy orthologs in [49] is comparable to the number of genes present in the LCBs for the ingroup taxa presented here (Table 1). Future work might ask whether the sets of orthologs are similar for these two data-sets and how these two different approaches (ortholog identification vs. unannotated homology detection) might complement each other to best utilize whole genome sequence data.

### Subset trees

It is interesting that none of the trees that resulted from analysis of each LCB separately produced the same topology as the genome tree, even though there are relatively few taxa, and that the randomly sampled data-sets all produced the genome-tree topology, even though some of the LCBs were longer than some of the randomly sampled data-sets. Sometimes *Shewanella *was not monophyletic in individual LCB trees. What the results highlight, is that with a genome approach, which does not only focus on particular genes of interest, one is able to discern a unique phylogenetic signal. The random data-set tree results demonstrate that localized LCB signal might be a factor, even when an LCB contains 25 or more genes. One might expect that sampling nucleotides across all LCBs produces a signal closer to the optimum than does focusing on parts of the genome with putatively different histories. The surprising insight here was that 20,000 bp was enough to see this effect, for the present taxon sampling. It should also be noted that Mauve provides a kind of filter, in that the LCBs consist of those parts of the genome that have passed tests of similarity and co-linearity. The 3 Mbp data-set, based on LCBs common to all taxa, represents a very complete, ideal data-set; approximately one-third of the genome (spread over the whole genome; Figure 1) for which all taxa have data. This is perhaps part of the reason that 20,000 bp is enough to recover the genome tree topology, in this case. As suggested by a reviewer, the possibility exists that 20,000 bp is simply enough for the model mis-specification artifacts caused by mixed-tree signals or incorrect substitution models to consistently yield an incorrect topology. This explanation would require, however, that all LCBs, or all LCBs except one also had this problem because they all produce different topologies that are not congruent with the genome tree.

For the seven-gene tree (Figure 3B), there is generally high bootstrap support, but there are only two ingroup taxa that retain the same sister-group relationships that are also present in the genome tree (Figure 2). The genes chosen for the analysis were the same (minus 16S rRNA) as those chosen for a phylogenetic analysis for Vibrionaceae, the most closely related family to Shewanellaceae [46]. Even the four *S. baltica *strains appear scattered across the tree. The two large clades, which are consistent across all other trees, containing (*S. woodyi*, *S. sediminis*, *S. piezotolerans*, *S. halifaxensis*, and *S. pealeana*) and (*S. baltica*, *S. putrefaciens*, *S. sp. W3-18-1*, *S. sp. MR-4*, *S. sp. MR-7*, *S. sp. ANA-3*, and *S. oneidensis*) are not present in the seven-gene tree. *C. psychrerythraea*, a putative outgroup taxon is nested deeply within the ingroup in the seven-gene tree.

### 16S rRNA investigation

The fact that Mauve does not generate a hypothesis of positional homology for any single copy of 16S rRNA even though all copies are very similar speaks to the challenges that occur when dealing with multiple gene copies. The pattern of gene arrangement flanking instances of 16S rRNA is not enough to assign a hypothesis of positional homology. There is simply too much rearrangement to have confidence in such a hypothesis. The taxonomic relationships suggested by the all-copy 16S rRNA tree from TNT (Figure 4) are different than those suggested by the genome tree (Figure 2). Nodes of congruence are highlighted in Figure 4. There are many similarities between the all-copy 16S rRNA tree and the genome tree, however: *Shewanella *is monophyletic, *S. sp. MR-7*, *S. sp. MR-4*, *S. oneidensis*, *S. sp. ANA-3 *are monophyletic, *S. halifaxensis *and *S. pealeana *are sister, *S. denitrificans *and *S. frigidimarina *are sister, *S. sp. W3-18-1 *and *S. putrefaciens *form a clade, *S. piezotolerans*, *S. halifaxensis *and *S. pealeana *form a clade, *S. sediminis *and *S. woodyi *are sister, *Al. macleodii *and *C. psychrerythraea *are sister. The 16S rRNA copy tree from RaxML shared many nodes in common with the 16S rRNA tree from TNT, but found the *Al. macleodii *and *C. psychrerythraea *clade within *Shewanella *and did not find *S. denitrificans *and *S. frigidimarina *as sister. The fact that the TNT and RaxML trees are not completely congruent is not particularly surprising given that there are few parsimony informative characters in 16S rRNA and that there is no expectation that a tree-like branching pattern exist for these gene copies. For the TNT run, the gaps were treated as a fifth state, so that a gap can be informative. RaxML treats gaps as missing data, so that might also account for the differences.

Haggerty *et al*., [50] constructed a similar 16S rRNA copy tree, but did so for 17 species across four genera: *Escherichia*, *Shigella*, *Yersinia*, and *Salmonella *(also Gammaproteobacteria). They found the backbone of this tree, i.e. the separation of genera, conforming to their taxonomic expectations. They did not find widespread monophyly of copies at the species level, however. The tree shown in Figure 4, based on the present analysis, does show monophyly for many species. The following taxa formed monophyletic groups of 16S rRNA copies: *C. psychrerythraea*, *Al. macleodii*, *S. loihica*, *S. woodyi*, *S. sediminis*, *S. pealeana*, *S. amazonensis*, *S. putrefaciens*, *S. oneidensis*, *S*. sp. ANA-3, *S. denitrificans*, *S. frigidimarina*. These taxa formed paraphyletic groups: *S. halifaxensis *(*S. pealeana *nested inside), *S*. sp. W3-18-1 (*S. putrefaciens *nested inside). The following taxa had gene copies scattered among those of other taxa, *S*. sp. MR-7, *S*. sp. MR-4, *S. baltica *OS155, *S. baltica *OS185, *S. baltica *OS195, *S. baltica *OS223.

There is no expectation that this is the actual evolutionary history of these gene copies, or that it should provide the correct relationships among taxa, because multiple gene copies cannot all be orthologous to one another, and are homologous only at the level that there was one initial copy of 16S rRNA, but that is not informative at this phylogenetic level of inquiry. It was merely an exercise to see if the copies from one species group more closely with the other copies for that species or with copies from another species. The former is most often the case. The notable exceptions are the *S. baltica *strains, which are classified as strains of the same species and the *S. sp*. MR-4 and *S. sp*. MR-7. *S. sp*. MR-4 and MR-7 were isolated from different depths of the Black Sea [14]. It follows that these may have been separate lineages for a much shorter period of time than the other taxa included in the analysis, or that there continues to be gene exchange. The major pattern, that of monophyly of within-species copies, might be explained in multiple ways. Haggerty et al., 2009 suggest that such a pattern might reflect "homogenization" of 16S rRNA copies such that each species has its own unique suite of copies of the gene. This is also known as concerted evolution [51]. The question remains as to why Haggerty et al., 2009 do not recover a concerted evolution type pattern, even though they consider taxa not so distantly related to *Shewanella *and with similar 16S rRNA copy numbers. It is possible that because they included only 17 species covering all 4 genera, their taxon sampling was too sparse to recover the true pattern. They also removed parts of the 16S rRNA alignment that were "ambiguous", further lowering the number of characters, probably the informative characters, in their analysis.

Putting the issue of multiple copies aside, it is not surprising that any of the copies individually is able to group with its species, given that 16S rRNA sequences are a significant part of how prokaryotic species are defined. What is more important for evolutionary studies, however, is that because there are multiple copies, and because in the analysis presented here no one single copy among the sampled taxa is found to be positionally homologous, as well as the whole genome topology and the all-copy 16S rRNA topology do not agree, 16S rRNA should not be considered a reliable marker for positing evolutionary relationships. Whether it performs well diagnostically is unrelated to this question.

## Conclusions

Recent phylogenetic studies have been published that rely on many more gene loci than has been customary in the past [*e.g*. 52-54]. These studies also begin to ask and answer questions regarding the number of genes sufficient to obtain the 'true' tree. Gene-tree concordance methods have also been adopted by many to this end [55,56]. The study presented here, which takes a different approach, can also begin to add to this discussion. Here, it is shown that unannotated whole genome data can provide excellent raw material for generating hypotheses of historical homology, which can be tested with phylogenetic analysis and compared with hypotheses of gene function. The future possibilities include the ability to quantify lateral gene transfer and gene tree effect, track changing function over gene history and find segments of co-evolving DNA. It is through the combination of methods of phylogenetic systematics and comparative genomics that we can best use the whole genome data to reconstruct the histories of genes, genomes, and taxa.
